# Exploring the sustained impact of the Mindfulness-Based Stress Reduction program: a thematic analysis

**DOI:** 10.3389/fpsyg.2024.1347336

**Published:** 2024-07-19

**Authors:** Meltem Yavuz Sercekman

**Affiliations:** Brunel Business School, Brunel University London, London, United Kingdom

**Keywords:** long-term impact, Mindfulness-Based Stress Reduction (MBSR), mindfulness-based intervention (MBI), Mindfulness practice, qualitative research, semi-structured interviews, thematic analysis

## Abstract

**Introduction:**

This study aimed to explore the time-varying impact of the Mindfulness-Based Stress Reduction (MBSR) program, specifically examining its effects on various variables 3 months, 1 year, and 3 years after program completion. Additionally, the study aimed to identify the barriers and facilitators in maintaining mindfulness practice over time and the preferred mindfulness practices among participants in three distinct time groups.

**Methods:**

The study utilized a qualitative research design, conducting semi-structured interviews with 45 participants who had completed the 8-week MBSR program at different time points. Thematic analysis was employed to analyze the qualitative data obtained from the interviews, allowing for the identification of key themes and patterns.

**Results:**

The findings revealed that the effectiveness of the MBSR program varied at different times and across different variables. Immediately after completing the program, participants experienced a significant decrease in stress levels and an increase in awareness. One year later, the program continued to have positive effects on inner calm, coping mechanisms, and relationships. Three years after completing the program, its long-term impact was observed in the adoption of a mindful lifestyle, increased compassion and kindness, and ongoing personal growth.

**Discussion:**

The study highlights the transformative potential of the MBSR program beyond short-term symptom relief. The long-term effects observed in overall wellbeing emphasize the sustained efficacy of mindfulness-based interventions. The identified barriers and facilitators in maintaining mindfulness practice provide insights for program implementation and individual engagement. By understanding the long-term impact and preferences of participants, tailored interventions can be developed to maximize the benefits of the MBSR program for individuals over time.

## 1 Introduction

Mindfulness-Based Stress Reduction (MBSR) is a program developed by Jon Kabat-Zinn in the late 1970s. As a group program, it combines elements of mindfulness meditation, Hatha yoga and body awareness practices to discover stress triggers and unhelpful automatic stress reactions to help individuals manage stress, and improve overall wellbeing (Kabat-Zinn, 1982). The standardized 8-week program aims to cultivate non-judgmental awareness of the present moment, enhance self-compassion, enabling individuals to respond to stressors with greater clarity and resilience (Kabat-Zinn et al., 1985; Kabat-Zinn, 2003). Participants engage in structured weekly meetings, each lasting ~2 to 2.5 h, over a period of 8 weeks. These sessions follow a standardized curriculum (Table 1). Additionally, a 5-h silent retreat is conducted during the sixth week. The weekly gatherings consist of both formal and informal mindfulness exercises, followed by group discussions. Participants are also provided with audio-guided meditation and exercises through recordings, complemented by a comprehensive handbook. In addition to attending these sessions, participants are encouraged to incorporate mindfulness practices into their daily routines through daily homework.

**Table 1:** The 8-week MBSR program.

**Week**	**Key theme**	**Key practices**	**Weekly homework**
1	Exploring mindfulness	• The raisin exercise (IF) • Body scan (F)	• Body scan (F) • Nine-dot exercise (IF) • Mindful eating (IF)
2	How we perceive the world and ourselves?	• Body scan (F) • Mindful seeing exercise (IF) • Sitting meditation with breath awareness (F)	• Body scan (F) • Sitting meditation with breath awareness (F) • Journaling—Pleasant moments calendar (IF)
3	Being at home in our own body	• Hatha yoga (F) • Sitting meditation with breath awareness (F)	• Body scan (F) • Yoga (F) • Journaling—Unpleasant moments calendar (IF)
4	What is stress?	• Hatha yoga (F) • Body scan (F)	• Body scan (F) • Sitting meditation (F) • Hatha yoga (F)
5	Stress reaction/mindful response	• Mindful hearing exercise & the practice of choiceless awareness (F)	• Sounds and thoughts meditation (F) • Journaling—Challenging communication calendar (IF)
6	Mindfulness communication	• Sitting meditation (F) • Mindful listening practice (IF)	• Body scan (F) • Hatha yoga (F)
Retreat day	Day of silence	• Body scan (F) • Walking meditation (IF) • Sitting meditation (F) • Silent eating (IF) • Metta meditation (F)	
7	Taking care of ourselves	• Standing body scan (F) • Sitting meditation (IF) • Mountain meditation (F)	• Sitting meditation (F) • Body scan (F) • Hatha yoga (F)
8	Looking backward, going forward	• Body scan (F) • Sitting meditation (F) • Writing a letter to ourselves (IF)	

F, formal mindfulness practices; IF, informal mindfulness practices.

MBSR has gained popularity and recognition for its effectiveness in various settings, including healthcare, education, politics, sports, and workplace environments. Research on MBSR has demonstrated promising results in promoting wellbeing and reducing psychological distress (Frank et al., 2015; Ghawadra et al., 2019; Lomas et al., 2019). Moreover, mindfulness practices have been associated with decreased anxiety (Goldin and Gross, 2010; Vøllestad et al., 2011), burnout prevention (Kinnunen et al., 2020), reduced depression symptoms (Mathew et al., 2010), and improvements in attention and overall mental health (Solhaug et al., 2019). The benefits of mindfulness-based interventions extend beyond individual wellbeing, impacting interpersonal relationships, work-related outcomes, and quality of life (Skoranski et al., 2019; Chu and Mak, 2020; McNall et al., 2021; Yavuz Sercekman, 2023). The mechanisms underlying these benefits are thought to involve increased self-awareness, enhanced cognitive flexibility, and improved emotion regulation, resilience, and self-compassion (Lao et al., 2016; Zou et al., 2020). Additionally, research on MBSR has demonstrated positive effects on physical health outcomes (Grossman et al., 2004). Studies have indicated that MBSR can improve immune system functioning, cardiovascular health, blood pressure and cortisol levels, sleep quality, and reduce chronic pain (Carlson et al., 2007; Brand et al., 2012; Pascoe et al., 2017; Chen et al., 2020). Mindfulness practice can result in enduring changes in brain structure and function (Kral et al., 2018).

There is a growing interest in understanding the long-term impact of the program (Staffaroni et al., 2017; de Vibe et al., 2018; Ribeiro et al., 2018; van Dijk et al., 2022). Despite the growing body of evidence supporting the immediate and long-term benefits of MBSR, yet the sustainability of these benefits is less understood. The duration for which individuals continue to practice mindfulness techniques after a program ends, and the persistence of the benefits, are still unclear. It is necessary to investigate if the effects of MBSR offer temporary relief or result in long-term, sustainable changes that aid in continuous stress management and enhance wellbeing. Understanding this aspect is crucial to measure the full potential of MBSR and its role in fostering lasting improvements in mental and physical health. In addition, it is necessary to comprehend the varying effects of MBSR over time and gain insight into the diverse impacts of MBSR across different time groups to provide a comprehensive understanding of its overall influence. This allows us to understand that the benefits of MBSR may develop gradually and continue evolving beyond the immediate post-program period. It can also enable the creation of tailored interventions and empowers individuals to make informed decisions about their mindfulness practice. This knowledge can assist participants in setting realistic expectations and motivate them to continue their mindfulness practice, knowing that the full benefits may take time to emerge. Thus, this research investigates the effects of the MBSR program over time, specifically examining the impact on various variables 3 months, 1 year, and 3 years after program completion. Additionally, the study explores the barriers and facilitators in maintaining mindfulness practice over time and identifies the preferred mindfulness practices among participants in three distinct time groups.

## 2 Method

This study employed a qualitative research methodology, known for its ability to provide rich, context-sensitive, and nuanced insights into participants' subjective experiences. This method allowed for a thorough exploration of participants' perspectives, attitudes, and behaviors regarding the Mindfulness-Based Stress Reduction (MBSR) program over time. Although quantitative methods can deliver valuable statistical data on key variables like emotion regulation, mood, wellbeing, and quality of life, they may not fully capture the complexity of human experiences. Therefore, a qualitative approach was deemed more appropriate for this study. In this context, semi-structured interviews were conducted with participants who had completed the program at three distinct time points: 3 months (T1), 1 year (T2), and 3 years (T3) post-program completion. These intervals were deliberately selected based on theoretical and empirical considerations. The first time point, T1, reflects short-term effects and captures immediate changes and the initial integration of mindfulness practices into daily routines. The second time point, T2, represents a medium-term effect, allowing for the exploration of the program's impact once the novelty has worn off and the practices have been further integrated or potentially abandoned. Finally, T3 provides insight into the long-term sustainability of the practices and the program's enduring effects, offering a comprehensive understanding of the MBSR program's influence over an extended period. These intervals collectively provide a broad temporal spectrum for evaluating its effects at different stages, thus facilitating a thorough understanding of the program's longitudinal impact and the sustainability of mindfulness practice. Participants who had completed the program at other time intervals, such as 7 months post-completion, were not included in this study to maintain the consistency and clarity of the temporal stages under investigation.

The qualitative data obtained from the interviews were subjected to thematic analysis to identify key themes and patterns. Thematic analysis (TA) was chosen as the qualitative data analysis method for this study due to its suitability for exploring patterns and themes within a large dataset of interview transcripts (Clarke et al., 2015). The method is a widely used and versatile approach that offers several key strengths that make it a useful method for this study. First, the flexibility of thematic analysis was crucial for this research as it enable to adapt the analysis to the specific research questions and context of the study. The MBSR program is a complex intervention with multiple dimensions, and it is needed a method that could capture the diverse experiences and perspectives of the participants. Thematic analysis provided the flexibility to explore the data in an open and iterative manner, enabling the emergence of new themes and insights that might not have been anticipated at the outset of the study (Peel, 2020). This flexibility allowed to delve deeply into the participants' experiences and gain a comprehensive understanding of the sustained impact of the MBSR program over time. Second, thematic analysis offered the ability to capture the richness and depth of the participants' experiences. By analyzing their own words and narratives, TA ensured that their voices were central to the analysis (Braun and Clarke, 2006). This was particularly important for a study examining the long-term and time-varying effects of the MBSR program, as it allowed to gain insight into their subjective experiences and the ways in which mindfulness practices were integrated into their daily lives. Furthermore, thematic analysis provided a systematic yet flexible framework for theory development (Riger and Sigurvinsdottir, 2016; Terry et al., 2017). By allowing to develop concepts, constructs, and themes grounded in the data, TA facilitated the generation of insights and theoretical frameworks that could inform future research and practice.

The iterative process of coding and theme development in thematic analysis helped to identify patterns and connections within the data, leading to a deeper understanding of the mechanisms underlying the sustained benefits of the MBSR program. This theoretical grounding is essential for advancing the field of mindfulness-based interventions and developing effective strategies to support individuals in maintaining their mindfulness practice. These strengths of thematic analysis were instrumental in providing a comprehensive understanding of the sustained impact of the MBSR program and its practical implications for researchers and practitioners in the field of mindfulness-based interventions.

### 2.1 Participants

The study recruited individuals from Turkiye who had participated in an 8-week MBSR program. Purposive sampling selected participants from three distinct time groups. As the researcher has been teaching the MBSR program since 2019, she shared the research announcement on her social media platforms (LinkedIn and Instagram) and via newsletters to previous participants who had given consent. Other mindfulness teachers also amplified the recruitment effort by sharing the invitation on their accounts. Prospective participants expressed interest by responding via email. A pre-registration form assessed their eligibility. Participants included in the study were those who had completed the MBSR program either 3 months, 1 year, or 3 years ago. Individuals who completed the program outside these time frames or participated in other mindfulness-based interventions or teacher training programs after the MBSR were excluded. Ultimately, 15 individuals from each group were selected, totaling 45 participants. The sample was diverse in terms of age, gender, and background, enhancing the generalizability of the findings. Out of the 45 participants, 37 are employed full-time in various sectors, including healthcare and medical, education, finance and banking, manufacturing, retail and e-commerce, professional services and tourism, government and public administration, non-profit organizations, and construction and engineering. There are eight individuals who are currently not working due to reasons such as maternity leave, job searching, or recently leaving their jobs. Additional participant details are summarized in Table 2.

**Table 2:** Participant characteristics.

	**T1 (*n* = 15)**	**T2 (*n* = 15)**	**T3 (*n* = 15)**
Average age (*M*)	33.8	32.47	34.13
Female	12	14	13
Male	3	1	2
**Relationship status**			
Married/cohabiting	8	7	10
Single	7	8	5
**No of children**			
0	8	6	7
1>	7	9	8
**Education degree**			
Highschool	1	0	0
Bachelor's degree	8	6	9
Master's degree	5	7	6
PhD degree	1	2	0

M, mean; T1, 3 months later, T2, 1 year later, T3, 3 years later after completing the MBSR program.

### 2.2 Procedure

Semi-structured interviews were conducted in October 2023 with the participants to gather qualitative data on their experiences with the MBSR program and its effects. The interviews were conducted individually using Zoom or Teams and lasted ~45 min to 1 h. The interviews were audio-recorded with the consent of the participants, and detailed field notes were taken during and after each interview to capture non-verbal cues and contextual information.

### 2.3 Data analysis methods

In this study, a thematic analysis was used to analyze the qualitative data obtained from the interviews. The analysis followed a six-phase process: familiarization with the data, generating initial codes, searching for themes, reviewing themes, defining, and naming themes, and producing the final report (Braun and Clarke, 2006). Throughout the analysis process, MAXQDA, a software specifically designed for qualitative data analysis, was utilized to efficiently organize, and retrieve data. This software facilitated the management of the large amount of qualitative data and allowed for easy navigation between different segments of the interviews.

The analysis was conducted in a systematic and iterative manner to ensure the reliability and validity of the findings. First, the audio recordings of the interviews were transcribed verbatim, and the transcriptions were reviewed multiple times to gain familiarity with the data. This familiarization phase allowed for immersion in the participants' experiences and ensured a deep understanding of the context. Next, initial codes were generated by identifying patterns, concepts, and recurring ideas within the data. This involved a systematic and inductive process of labeling and categorizing the data. The codes were organized into potential themes by grouping together related codes. This phase allowed for the identification of meaningful patterns and connections within the data. The identified themes were then reviewed and refined through an iterative process of comparison and contrast. This involved revisiting the raw data and ensuring that the themes accurately captured the participants' experiences. The themes were defined and named to provide a clear and concise representation of the data. The final themes were used to interpret and present the findings in a coherent and meaningful manner.

It is important to note that as the researcher conducting the analysis, my active involvement in applying the thematic analysis approach played a crucial role in shaping the interpretation of the data. By immersing myself in the participants' narratives, actively coding and organizing the data, and making meaning out of the themes, I contributed to the analytic process. The findings of the thematic analysis were interpreted within the context of relevant scholarly fields, such as mindfulness-based interventions, psychological wellbeing, and coping mechanisms. By connecting the analysis to these fields, the interpretation of the findings was grounded in existing theoretical frameworks and contributed to the broader understanding of the sustained impact of mindfulness-based interventions, specifically the Mindfulness-Based Stress Reduction (MBSR) program. Overall, the thematic analysis process allowed for a rigorous exploration of the qualitative data, enabling the identification of key themes and patterns that shed light on the long-term and time-varying effects of the MBSR program.

## 3 Results

The findings of this study demonstrate different levels of effectiveness of the MBSR program among different time groups and variables. Additionally, the study investigated the barriers and factors that facilitate the maintenance of mindfulness practice over time. It also identified the most preferred mindfulness practices among participants from three different time groups: those who completed the MBSR program at 3 months (T1), 1 year (T2), and 3 years (T3) after program completion.

### 3.1 Impacts of the MBSR program across time groups and variables

**Figure 1:**
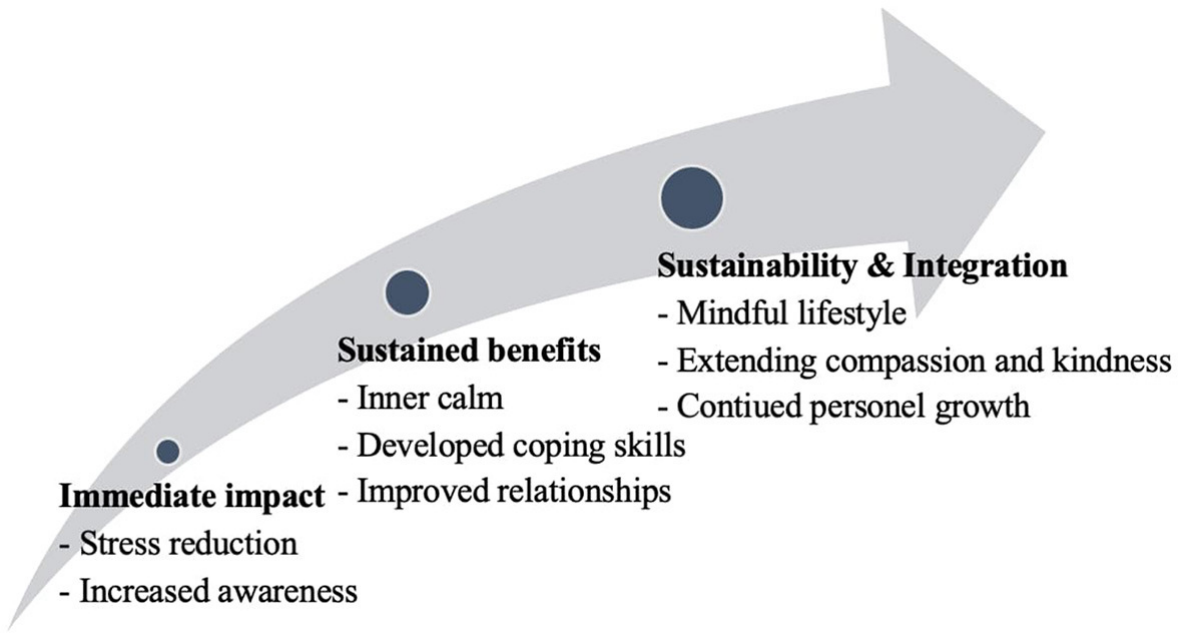
Time-varying impacts of the MBSR program across groups.

The 45 interviews yielded a total of 198 codes, which were categorized into 51 categories and further grouped into nine subthemes under three main themes (Figure 1). Thematic analysis of the qualitative data highlighted significant differences in the effects of the MBSR program among participants in three different time groups. The following themes emerged:

#### 3.1.1 Immediate impact (3 months after program completion)

The MBSR program appears to be most effective in reducing stress levels shortly after completion. Participants reported immediate benefits in stress reduction and increased awareness.

##### 3.1.1.1 Stress reduction

The participants who were involved in this group have reported experiencing significant reductions in their stress levels shortly after they completed the program. They described a state of increased relaxation and an enhanced ability to cope with the stressors they encounter in their daily lives. The program appears to have equipped them with effective stress management tools and strategies, resulting in an overall improved mental wellbeing.

P4, T1: “*I noticed a significant reduction in my stress levels after completing the program. I feel more relaxed and better equipped to handle the daily stressors that come my way.”*

##### 3.1.1.2 Increased awareness

Many participants acknowledged a significant increase in their awareness levels regarding their own thoughts, emotions, and physical sensations. They articulated a newfound understanding and attentiveness toward their mental and emotional state, as well as a heightened awareness of their bodily sensations. This increased awareness allowed them to be more present and mindful in their daily activities. They reported a noticeable improvement in their capacity to remain in the present moment, fully engaged and mindful, whether they were involved in work, interpersonal relationships, or personal pursuits.

P8, T1: “*I have become more aware of my thoughts, emotions, and body. I find myself being more present and mindful in my daily activities, which has brought a greater sense of clarity and awareness to my life.”*

#### 3.1.2 Sustained benefits (1 year after program completion)

The program's effects on reducing stress and increasing awareness seem to be long-lasting. Additionally, participants in this group reported experiencing positive effects on inner calm, coping mechanisms, and relationships.

##### 3.1.2.1 Inner calm

The participants in this group have consistently reported that the stress reduction effects of the program have been effectively maintained over an extended period. They underscored the enduring impact of the mindfulness practices imparted in the program, outlining how these techniques have been instrumental in helping them cultivate a deep sense of inner calm. These practices, they shared, have allowed them to manage their stress levels more efficiently, enabling them to lead more balanced and harmonious lives.

P1, T2: “*Even after a year of completing the program, I have noticed a significant reduction in my stress levels. The mindfulness practices have become a part of my daily routine and help me find inner calm.”*

P6, T2: “*The mindfulness techniques I learned during the MBSR program continue to help me reduce stress. I feel calmer and more centered, even in challenging situations.”*

##### 3.1.2.2 Developed coping skills

The participants in the study not only highlighted the development of effective coping mechanisms through mindfulness-based practices, but they also emphasized the continued utilization of these skills in their everyday lives. These coping mechanisms were not merely temporary solutions, but rather long-term strategies that they incorporated into their day-to-day routines. This resulted in a few significant benefits, as reported by the participants. They observed a noticeable reduction in symptoms related to anxiety and depression, and a marked improvement in their emotional regulation. Moreover, they also reported increased resilience, which played a crucial role in their ability to deal with stressful situations and bounce back from adversities.

P4, T2: “*The program taught me how to cope with stress in a healthier way. I find myself applying those mindfulness techniques and feeling more resilient.”*

P10, T2: “*On February 6, 2023, in an earthquake that occurred in 10 different provinces in Turkiye, I lost dozens of my relatives, and I was rescued from the rubble at the last minute. This situation deeply affected me, and I started to have frequent anxiety attacks. However, thanks to the knowledge and skills I learned from the program, I can manage these attacks, I can cope with the pains. I think the program has been very helpful to me in this regard.”*

##### 3.1.2.3 Improved relationships

Participants noted that the incorporation of mindfulness practices into their daily routines was a prominent factor in enhancing their social and personal interactions. They stated that these practices allowed them to cultivate a more profound understanding of their relationships and significantly improved their communication skills. This improvement was not just superficial, but it allowed them to connect on a deeper emotional level. They described experiencing increased levels of empathy, which resulted in a stronger and more meaningful bond with others. Their relationships became more enriched, and they felt more attuned to the needs and feelings of those around them.

P3, T2: “*Since completing the MBSR program, I have noticed a positive change in my relationships. I feel more empathetic toward others, and I am better able to put myself in their shoes. This has helped me communicate more effectively and build deeper connections with the people in my life.”*

P5, T2: “*The MBSR program has had a significant impact on my relationships. I have become more mindful of my interactions with others, which has improved my communication skills. I now listen more attentively and respond with greater empathy, leading to stronger and more meaningful connections.”*

#### 3.1.3 Sustainability and integration (3 years after program completion)

The MBSR program demonstrates its ongoing effectiveness in the long term, particularly in terms of sustainability and the integration of mindfulness practices into everyday life. Three years after completing the program, its long-term impact was observed in the embracing of a mindful lifestyle, increased compassion and kindness, and ongoing personal growth.

##### 3.1.3.1 Mindful lifestyle

Participants in this group integrated mindfulness practices into their daily routines in a sustainable manner. They regularly engaged in especially informal mindfulness exercises and applied a mindful approach to different activities. Participants reported a significant enhancement in their overall quality of life, leading to positive changes across multiple areas. Furthermore, the MBSR program notably promoted physical health, seen in improved sleep quality, more mindful eating habits, and increased energy levels.

P1, T3: “*It's been three years since I completed the program and during this time, I've noticed a significant improvement in my quality of life. My mood has generally been more positive, and I've seen changes in my physical health too - my sleep quality has improved because now I make more conscious choices before going to sleep. I am aware of the stressors that could disrupt my sleep and I was able to create a healthy morning and evening routine.”*

P9, T3: “*The mindfulness practices I learned during the MBSR program have become an integral part of my daily life. I now incorporate mindfulness into my everyday routines, such as mindful eating and mindful walking. It has brought a sense of presence and awareness to my life.”*

##### 3.1.3.2 Extending compassion and kindness

Three years after the program's completion, the participants greatly emphasized the development of compassion and kindness as significant and transformative outcomes of their experience. They provided intricate details of their personal transformation, where the journey was marked by a considerable increase in self-acceptance and the practice of self-care. They noted a paradigm shift in their self-perception, moving away from a state of self-criticism and negativity, toward a more forgiving and kinder view of themselves. This shift was not merely a change in self-perception, but a profound alteration of their self-identity, allowing them to cultivate a more positive outlook on life. Moreover, they reported a developed sense of heightened empathy and a deeper compassion for others. These changes were far more than personal growth, they were profound strides in their emotional intelligence, giving the participants a renewed perspective on their relationships. These emotionally significant changes did not only stay confined to their personal lives but seeped into their social interactions as well. They reported that the effects were far-reaching, positively impacting their interactions with others, ultimately leading to more fulfilling connections.

P8, T3*: “The journey I embarked on with the MBSR program has been transformative. It has allowed me to cultivate a deep sense of self-acceptance and compassion toward myself and others.”*

P2, T3: “*I have become more aware of my own emotions and reactions, allowing me to respond to others with greater kindness and patience.”*

##### 3.1.3.3 Continued personal growth

Participants emphasized the ongoing personal growth and transformation they experienced over 3 years. This growth extended beyond their professional lives to personal aspects as well. Participants noted an improved ability to navigate life's challenges, demonstrating resilience and adaptability. The growth experienced by the participants had a significantly positive impact on their lives, fostering greater self-assurance, satisfaction, and happiness.

P4, T3: “*I have noticed a significant growth journey throughout the three years. It has enhanced my ability to navigate life's ups and downs, fostering resilience and an improved sense of well-being. Here, what I have learned has been reflected in every aspect of my life. From being able to say no to someone, to knowing when to take a break, to when I need to distance myself from a relationship or workplace, I feel wiser about myself.”*

While the program provides immediate benefits in reducing stress levels, its effectiveness extends to the development of sustainable mindfulness practices and personal growth over time. Although the specific impacts may vary among the three groups, there are common positive experiences reported by participants. All three groups expressed noticeable reductions in stress levels after completing the program, leading to feelings of calmness, relaxation, and improved ability to manage daily stressors. Many participants also reported improvements in their mental health and wellbeing. These improvements included reduced symptoms of anxiety and depression, increased self-awareness, and improved emotional regulation. Participants highlighted the development of effective coping mechanisms and increased resilience as outcomes of the MBSR program, enabling them to better handle challenges and bounce back from setbacks. The program also had a positive impact on various aspects of their lives, such as relationships, work-life balance, and life satisfaction. Those who successfully integrated mindfulness practices experienced a heightened sense of self-awareness and deeper connections with themselves and others. They described increased presence, acceptance, and compassion in their daily lives and incorporated mindfulness practices into their routines, such as mindful eating, meditation, and mindful breathing exercises. The integration process involved adopting a broader perspective and cultivating a mindful approach to thoughts, emotions, and actions, ultimately enhancing self-awareness, and promoting overall wellbeing.

### 3.2 Understanding barriers and facilitators in maintaining mindfulness practice over time

The findings indicate that the barriers and facilitators to maintaining mindfulness practices can vary depending on the time elapsed after individuals complete the program (Table 3). However, it is important to note that the classifications of barriers and facilitators summarized here do not show a sharp distinction based on time groups. There are common experiences, as well as differences between the groups. For instance, while lack of time is mentioned in almost every group, group support stands out as a differentiating factor for the T1 group. In the T3 group, internal sources of motivation are emphasized as a dominant facilitator, while the need for an established routine emerges for T2.

**Table 3:** Barriers and facilitators in maintaining mindfulness practice.

	**Barriers**	**Facilitators**
T1	Lack of consistency	Group support
T2	Life events and transitions	Established routine
T3	Complacency	Intrinsic motivation

T1, 3–6 months later, T2, 1–2 year later, T3, 3 years later after completing the MBSR program.

#### 3.2.1 Barriers

##### 3.2.1.1 Lack of consistency

In the initial months after program completion, participants mostly face challenges in maintaining a consistent mindfulness practice due to competing demands and a decrease in initial motivation and enthusiasm. Participants in this group reported struggling to maintain a consistent mindfulness practice due to competing demands and a lack of time. They mentioned that the initial enthusiasm and motivation from completing the program sometimes waned over time.

##### 3.2.1.2 Life events and transitions

One year after program completion, participants in this group predominantly mentioned that major life events or transitions, such as job changes, relocation, or family responsibilities, sometimes disrupted their mindfulness practice. They reported that these external factors made it challenging to prioritize and allocate time for mindfulness.

##### 3.2.1.3 Complacency

Three years after program completion, participants usually may experience a sense of complacency, feeling that they have mastered mindfulness techniques and no longer need to invest as much effort. Participants in this group shared that after integrating mindfulness into their lives for an extended period, they occasionally faced a sense of complacency. They described feeling that they had mastered the techniques and no longer needed to invest as much effort into their mindfulness practice.

#### 3.2.2 Facilitators

##### 3.2.2.1 Group support

In the initial months, participants find group support crucial in maintaining motivation and commitment to their mindfulness practice in a large extent. Participants in this group highlighted the importance of the group support they received during the MBSR program. They reported that staying connected with fellow participants and engaging in mindfulness-related discussions helped them stay motivated and committed to their practice.

##### 3.2.2.2 Established routine

One year after program completion, participants generally emphasize the importance of establishing a regular mindfulness practice routine, treating it as a non-negotiable activity in their daily schedules. Participants in this group emphasized the value of establishing a regular mindfulness practice routine. They shared that incorporating mindfulness into their daily schedule and treating it as a non-negotiable activity helped them maintain consistency.

##### 3.2.2.3 Intrinsic motivation

Three years after program completion, participants demonstrate a strong intrinsic motivation, driven by the long-term benefits they have experienced, to sustain their mindfulness practice. Participants especially in this group expressed a strong intrinsic motivation to continue their mindfulness practice. They reported that the long-term benefits they experienced, such as improved wellbeing and resilience, served as powerful motivators to sustain their practice.

### 3.3 Most preferred mindful practices among three groups

Understanding the mindful practices that are most preferred, and the daily time devoted to formal practices at different stages of the MBSR program can help in developing customized mindfulness interventions and providing support to individuals for maintaining their mindfulness practice over time. The analysis identified that breath awareness was the most preferred mindful practice among participants in all three groups (Table 4). Participants reported a strong preference for breathing awareness as their primary mindful practice. They emphasized that focusing on their breath was calming, grounding, and helped cultivate present-moment awareness while reducing stress. Sitting meditations were also commonly practiced, with a slightly higher frequency among participants from other time groups. This suggests that sitting meditations were initially emphasized during the program and continued to be valued by participants in the short term.

**Table 4:** Regular preferred mindfulness practices.

	**T1**	**T2**	**T3**
Body scan	3	2	0
Breath awareness	14	10	11
Sitting meditations	9	6	4
Hatha yoga practices	4	3	1
Loving-kindness meditation	3	1	1
Mountain meditation	0	1	2
Informal mindfulness practices	14	13	12

M, mean; T1, 3 months later, T2, 1 year later, T3, 3 years later after completing the MBSR program.

The study found that participants showed less engagement with other formal mindfulness practices like body scan, Hatha yoga, mountain meditation, and loving-kindness meditation. However, they consistently practiced informal mindfulness practices, such as incorporating mindfulness into daily activities. It is worth noting that 36 out of a total of 45 participants from all three groups emphasized the importance of mindfulness integration into daily life. They reported integrating mindfulness into various activities, such as eating, walking, and listening, as practical ways to maintain a continuous state of mindfulness throughout the day. This integration process involves adopting a broader perspective and cultivating a mindset that embraces mindfulness in various aspects of daily life. Participants described it to enhance self-awareness, self-compassion, promote overall wellbeing, and foster meaningful connections with themselves and others.

Participants were also asked about the daily time they spent on formal mindfulness practices. Based on the responses, it was observed that those who completed the program 3 months ago spent an average of 27.07 min, those who completed it 1 year ago spent 15.53 min, and those who completed it 3 years ago spent 8.67 min.

## 4 Discussion

The findings of this study indicate that the MBSR program reduces stress levels, promotes mental health recovery, improves coping mechanisms, and has a positive and sustained impact on various aspects of quality of life, consistent with previous research (e.g., de Vibe et al., 2018; Wen et al., 2021; van Dijk et al., 2022). The integration of mindfulness skills from the program teachings into daily life also emerged as a common theme among the participants, demonstrating the sustainability and practical applicability of the program teachings. Furthermore, the positive impact on areas such as relationships and work-life balance indicate that mindfulness practices could have broad effects beyond individual wellbeing.

Thematic analysis identified key barriers to sustaining mindfulness practice: lack of consistency, life events and transitions, and complacency. On the other hand, group support, an established routine, and intrinsic motivation were considered facilitators in sustaining mindfulness practice. Similarly, Nguyen et al. (2022) found participation and commitment to the practice and understanding of mindfulness as key enablers. These findings underscore the dynamic nature of sustaining mindfulness practice over time. The initial challenges of consistency and external factors are gradually replaced by routines and intrinsic motivation as individuals incorporate mindfulness into their lives. According to another previous study, these processes, like developing a mindfulness habit and perseverance, evolve as participants report improved wellbeing, changes in self-compassion, and a greater sense of control over thoughts (Banerjee et al., 2017). As a result, understanding these barriers and facilitators can guide the development of strategies to support individuals in sustaining mindfulness practice. For example, offering ongoing group support beyond program completion, emphasizing the importance of routine, and fostering intrinsic motivation by reminding them of long-term benefits can increase the sustainability of mindfulness practice.

Overall, the findings indicate that different stages of the MBSR program are associated with varying preferences for mindful practices. For example, breathing awareness and sitting meditations are commonly preferred early on as formal mindfulness practices. Participants emphasized the benefits of directing their attention to the sensations of the breath, which anchored them in the present moment and fostered inner peace. This suggests that participants found breath awareness to be a beneficial and accessible technique for cultivating mindfulness. It is worth noting that the frequency of engagement in breath awareness remained relatively consistent across the three different time groups, indicating the sustained popularity of this practice. Additionally, participants from all groups expressed a continued preference for sitting meditation as one of their primary mindful practices. They stated that they enjoyed systematically bringing their attention to different parts of their body, noticing sensations, breath, sounds, thoughts, and emotions. The research discovered that participants demonstrated lower levels of involvement in other formal mindfulness practices, such as body scan, Hatha yoga, mountain meditation, and loving-kindness meditation. One potential justification for the lower levels of engagement in these mindfulness practices among the participants could be the challenges they faced in integrating these practices into their daily routines. While the study focused on the long-term impact of the MBSR program, it is important to recognize that maintaining a consistent mindfulness practice requires ongoing effort and commitment. Factors such as time constraints, competing priorities, and difficulty in establishing a routine may have hindered participants from fully engaging in formal mindfulness practices. Additionally, individual differences in preferences and personal circumstances could have influenced the extent of their engagement in these practices. It is worth noting that even with lower levels of involvement in some formal practices, participants may still have experienced the benefits of mindfulness through other informal practices or the application of mindfulness principles in their daily lives. The other potential explanation for the most decreased preference for body scan meditation could be related to the nature of the practice itself. Body scan meditation requires individuals to bring their attention to physical sensations in the body, which can be challenging for some participants. It may require a higher level of focus and concentration compared to other mindfulness practices, such as breath awareness. Additionally, body scan may also bring up uncomfortable sensations or emotions that some participants may find difficult to tolerate. This practice involves exploring the body and noticing any areas of tension or discomfort, which can be triggering for individuals who have a history of trauma or body-related issues. Furthermore, personal preferences and individual differences in mindfulness practice may also contribute to the decreased preference for body scan meditation. Some participants may naturally resonate more with other mindfulness practices and find them more enjoyable or beneficial for their wellbeing. It is important to note that the decreased preference for any formal mindfulness meditation does not imply its ineffectiveness or lack of benefits. Different mindfulness practices can have varying effects on individuals, and what may be unfavored by some participants may still be valuable for others. Mindfulness is a highly individualized practice, and it is crucial to explore and find the techniques that work best for everyone's unique needs and preferences. As a matter of fact, as participants progress in their mindfulness journey, they tend to adopt and integrate mindfulness into daily life becomes an essential aspect of long-term mindfulness practice as participants advance and sustain informal mindfulness practices throughout the day. This emphasizes the importance of integrating mindfulness into daily life and acknowledging the value of informal practices in sustaining mindfulness. These findings highlight the need to provide a variety of mindfulness exercises to accommodate individual preferences and needs in mindfulness programs.

The study also revealed that daily time spent on formal mindfulness practices varied across different completion time groups. Those who completed the program most recently spent the longest time, followed by those who completed it 1 year ago, and those who completed it 3 years ago. The differences in time spent suggest a gradual decrease in daily practice over time. One possible reason for the variation in time devoted to formal mindfulness practices among participants at different time points is the concept of habit formation. It is likely that participants who recently completed the program are still in the process of establishing a consistent mindfulness practice and therefore dedicate more time to formal practices. Over time, individuals may become more skilled at incorporating mindfulness into their daily lives and require less time for formal practices while still reaping the benefits. Furthermore, participants who completed the program several years ago may have a deeper understanding of mindfulness and its integration into their routines, enabling them to effectively practice mindfulness in shorter durations. It is important to consider that individual preferences and personal circumstances can also influence the time allocated to formal practices.

## 5 Limitations and future directions

It is important to acknowledge some limitations of this study. First, while efforts were made to ensure diversity within the relatively small sample, the findings may not fully represent the larger population of individuals who have completed the MBSR program. Second, although the qualitative nature of this study provides rich insights into participants' experiences and perceptions, the reliance on self-reported data from interviews, which introduces the possibility of response biases. Participants may have provided socially desirable responses or had difficulty accurately recalling their experiences. Therefore, further research using quantitative measures is needed to validate and generalize these findings. Furthermore, the absence of a control group limits the ability to attribute the observed effects solely to the MBSR program. Future studies could benefit from including a control group and using experimental methods to establish a more robust causal relationship.

Further research could explore strategies to address the barriers and enhance engagement in structured mindfulness practices to optimize the long-term effects of MBSR. Future research could also investigate the reasons behind the decreased preference for body scan meditation and explore alternative mindfulness practices that may be more appealing and effective for individuals who struggle with this specific practice. This could enhance overall engagement and adherence to mindfulness-based interventions, allowing individuals to fully benefit from incorporating mindfulness into their lives. Considering these limitations, future research should address these methodological challenges and further explore the temporal dynamics of the MBSR program's effects. This will provide a deeper understanding of the program's efficacy, identify the mechanisms underlying its effectiveness, and refine its implementation for optimal outcomes.

## 6 Conclusion

Overall, this research contributes to the growing body of evidence supporting the effectiveness of the Mindfulness-Based Stress Reduction (MBSR) program and provides valuable insights for both researchers and practitioners in the field. This research addresses a gap in the existing literature by examining the long-term and time-varying effects of the MBSR program. While previous studies have primarily focused on immediate outcomes (e.g., Frank et al., 2015; Hill et al., 2017; Querstret et al., 2020; Chang et al., 2021), this study expands upon the current knowledge by comparing different time groups that have completed the program and investigating the continued benefits over a period of 3 years. The findings underscore the importance of sustained mindfulness practice in promoting wellbeing and offer practical implications for the development of future interventions. The utility of MBSR as a toolkit for both clinical and non-clinical populations lies in its versatility and broad applicability. It provides effective strategies for managing stress and promoting mental health recovery, which are relevant to a wide range of individuals regardless of their clinical status. Moreover, MBSR can be tailored to suit the specific needs and preferences of different individuals, making it a flexible and personalized mental health management approach. For clinical populations, MBSR can complement traditional therapeutic interventions. Based on the findings of this study, MBSR can also be a comprehensive and valuable tool for non-clinical populations.

## Data availability statement

The raw data supporting the conclusions of this article will be made available by the authors, without undue reservation.

## Ethics statement

The studies involving humans were approved by Brunel University London College of Business, Arts and Social Sciences Research Ethics Committee. The studies were conducted in accordance with the local legislation and institutional requirements. The participants provided their written informed consent to participate in this study.

## Author contributions

MYS: Writing – original draft, Writing – review & editing.
